# Regeneration of sensory nerve branches in extraction socket and surrounding alveolar bone in rat: immunohistochemical observation of the axon and myelin sheath changes

**DOI:** 10.1007/s10266-022-00772-y

**Published:** 2022-12-03

**Authors:** Wataru Kakuta, Satoru Matsunaga, Yuto Otsu, Kei Kitamura, Shinichi Abe, Yasutomo Yajima, Hideshi Sekine

**Affiliations:** 1 Department of Oral and Maxillofacial Implantology, Tokyo Dental College, 2-9-18 Kandamisaki-Cho, Chiyoda-Ku, Tokyo, 101-0061 Japan; 2 Oral Health Science Center, Tokyo Dental College, 2-9-18 Kandamisaki-Cho, Chiyoda-Ku, Tokyo, 101-0061 Japan; 3 Department of Anatomy, Tokyo Dental College, 2-9-18 Kandamisaki-Cho, Chiyoda-Ku, Tokyo, 101-0061 Japan; 4 Department of Histology and Developmental Biology, Tokyo Dental College, 2-9-18 Kandamisaki-Cho, Chiyoda-Ku, Tokyo, 101-0061 Japan; 5 Department of Fixed Prosthodontics, Tokyo Dental College, 2-9-18 Kandamisaki-Cho, Chiyoda-Ku, Tokyo, 101-0061 Japan

**Keywords:** Extraction socket, Myelin sheath, Axon, Regeneration of sensory nerve ending, Immunohistochemical staining

## Abstract

The purpose of this study was to investigate the process and derivation of the distribution of the sensory nerves that appear in the extraction socket and surrounding alveolar bone following tooth extraction. The right mandibular first molar of rats and periodontal ligament were extracted as a single mass, and the mandible was harvested after days 1, 3, 5, and 7 after extraction. Serial sections of 7 µm thickness were prepared for the proximal root (Section A), buccolingual root (Section B), and centrifugal root (Section C) of the first molar. H–E staining and immunohistochemical staining with anti-S100 antibody and anti-NF-L antibody were carried out. The presence of nerve fiber bundles in the blood clot was already evident on post-extraction day 3, and on post-extraction day 7. On day 3, the number of axons in Sections B and C had greatly decreased, indicating that, after extraction, the connection between peripheral nerve tissue and the trigeminal ganglion was temporarily markedly reduced in the region of the alveolar branch. Although the myelin sheaths were regenerating on day 5, the majority of the axons of the alveolar branches extending from the inferior alveolar nerve were seen to be extremely thin and scattered, despite their further regeneration. The above results suggest that the newly myelinated nerves are actually derived from the bone marrow to the extraction socket, so few nerves, rather than being derived from the alveolar branches that had innervated the extracted tooth.

## Introduction

Tooth pulp, which together with dentin is completely enclosed within the teeth, senses all external stimuli as pain, and primary afferent fibers passing through the apical foramen are connected to the cell bodies of the trigeminal ganglion via the inferior alveolar nerves [1, 2]. The periodontal ligament also contains Ruffini-like nerve endings that sense tooth movements, as well as free nerve endings, and these control occlusal pressure by sending sensory information to the central nervous system via the trigeminal nerves [3, 4]. Ordinarily, the bones of the trunk and limbs are innervated by autonomic nerves that mainly regulate local blood flow in association with vascular smooth muscle cells, and although there have been reports of organ linkage induced by the very small number of sensory nerves that also innervate bone [5, 6], almost few studies have addressed the association of these with pain and other clinical symptoms. In contrast, alveolar bone is very densely innervated by sensory nerves, mainly in the teeth and periodontal ligaments, and the addition of autonomic nerves means that it contains a nervous system that can be described as alveolar bone-specific [7–10]. In these studies, Immunohistochemical staining was performed using an anti-S100 antibody to identify myelin sheaths [10] and an anti-NF-L antibody to identify axons [7].

However, when a tooth is extracted, the pulp and periodontal ligament are completely severed and destroyed. Noma conducted a detailed study of the healing of the extraction socket and the surrounding periodontal tissue after tooth extraction from the perspective of vascular network reconstruction [11, 12]. According to them, after the extraction socket has been filled with the residual blood clot, newly generated blood vessels extend from the remaining periodontal ligament to form a network, while new bone is generated as the blood clot is absorbed. Gunjigake *et al.* studied the effect of tooth extraction on the cell bodies of the trigeminal ganglion and reported that a retrograde response occurs comparatively rapidly post-extraction, which may affect the healing of the extraction socket [13]. However, according to the theoretical mechanism of nerve regeneration, injury to nerve fibers causes retrograde degeneration (primary degeneration) extending toward the central nervous system and Wallerian degeneration (anterograde degeneration) extending toward the nerve endings [14, 15]. This impairment is believed to start from a few minutes to a few hours after extraction, with retrograde degeneration evident as retroplasia of the axon and myelin sheath to a point several nodes of Ranvier behind, after which numerous regenerating axons start to sprout from this point. The tips of the regenerating axons have a ball shape called the “growth cone” and engage in amoeboid movement that takes approximately 1 week to reach the site of injury, a period called the “initial delay” [16, 17]. That is, in light of the mechanism of nerve regeneration, axons should always regenerate from a point closer to the central nervous system than the site of nerve injury, with new axons elongating toward the blood clot inside the extraction socket, and this process requires some time. In fact, extraction and other causes of peripheral nerve injury reportedly cause major structural, biochemical, and physiological changes to primary sensory neurons [18–21], and further investigations of the extraction socket and its surrounding alveolar bone are required.

The objective of the present study was to investigate the process and derivation of the distribution of the sensory nerves that appear in the extraction socket and surrounding alveolar bone following tooth extraction, by conducting an analysis of retrograde changes and the subsequent process of regeneration of the alveolar branch of the inferior alveolar nerve when this has been severed by tooth extraction.

## Methods

### Animals

The experimental animals were 4-week-old, male Wistar rats (*n* = 20) each weighing approximately 250 g. These rats were divided into a control group (non-extraction group, *n* = 4) and experimental groups (samples collected 1, 3, 5, and 7 day post-extraction, each: *n* = 4). They were placed in plastic cages under a 12-h light/dark cycle, and the room temperature was checked daily. They had free access to chow and water throughout the experimental period.

### Surgical procedures

The rats were reared normally for the week prior to extraction. They were placed under general anesthesia by intraperitoneal administration of a combination anesthesia comprised of medetomidine hydrochloride (0.75 mg/kg, Nippon Zenyaku Kogyo Co., Ltd., Fukushima, Japan), midazolam (midazolam, 4.0 mg/kg, Sand Co., Tokyo, Japan), and butorphanol tartrate (5.0 mg/kg, Meiji Seika Pharma Stocks Co., Tokyo, Japan) was used. Atipamezole hydrochloride (1.5 mg/kg; Kyoritsu Seiyaku, Tokyo, Japan) was intraperitoneally injected as an anesthetic antagonist. The right mandibular first molar (M1) of each experimental animal was extracted, so that the tooth and the periodontal ligament were removed in a single block. It was confirmed that the periodontal ligament around M1 had been removed, after which the rats were sacrificed on days 1, 3, 5, or 7, respectively, and the mandible was harvested. The animal experiment was performed with the approval of the Tokyo Dental College Animal Experiment Committee (Ethics Application No.: 213303). In rigorous conformity with the recommendations of the Helsinki Declaration, distress was alleviated by minimizing invasiveness in the treatment of the experimental animals.

### Histological observations

**Fig. 1:**
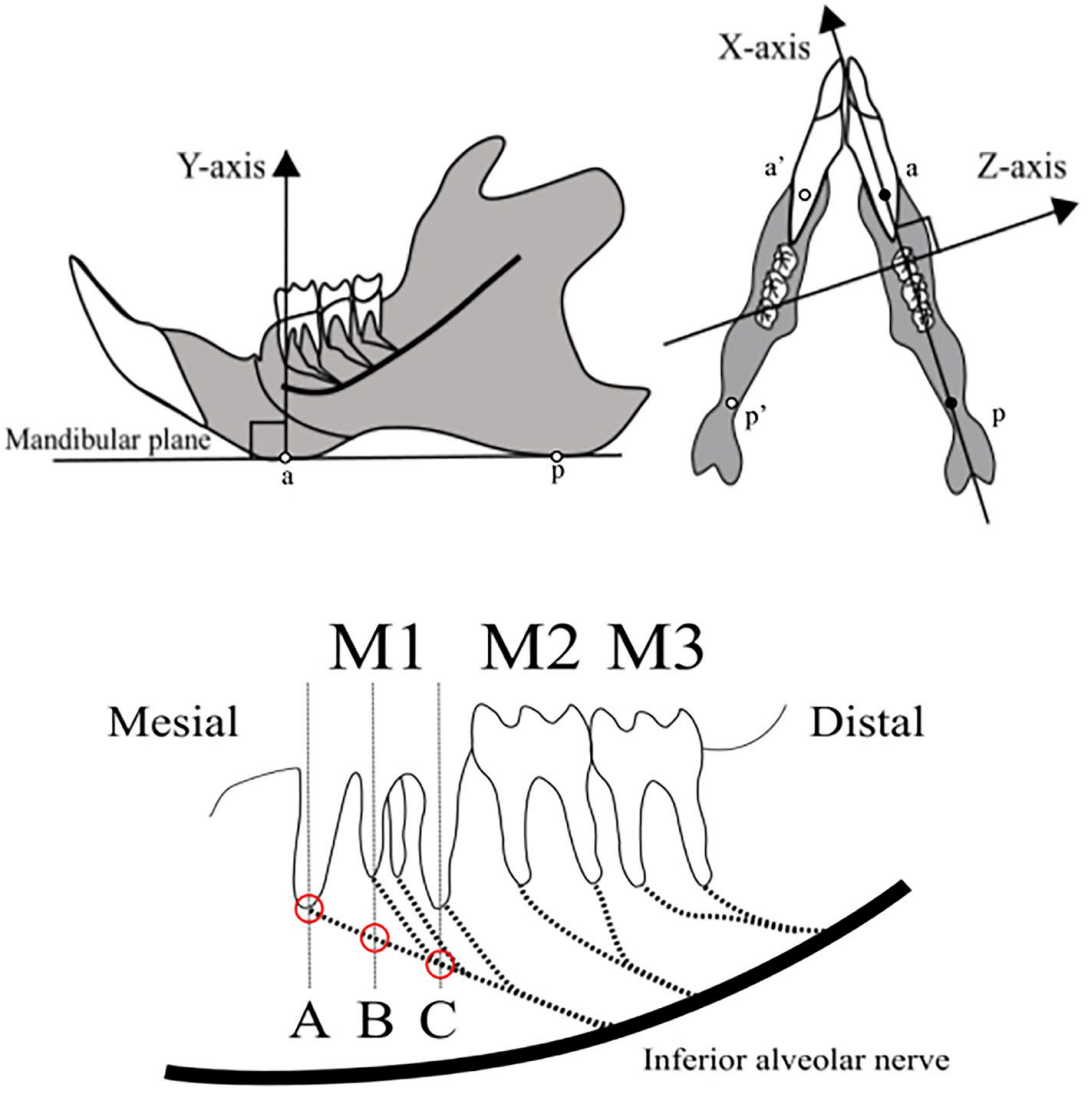
Designation of ROIs and three reference axes. The *X*-axis is defined as the extension of the line joining the lowest point of the thickened area of the anterior mandible (**a**) and the lowest point of the thickened area of the posterior mandible (**b**), and the *Z*-axis as the axis perpendicular to the *X*-axis on the plane passing through points a, a’, p, and p’ (the XZ plane). The *Y*-axis is defined as the axis orthogonal to these two axes. The ROIs are designated as the center of the mesial root (**A**), the center of the buccolingual root (**B**), and the center of the distal root (**C**) of the first molar

After harvesting, all samples were fixed in 4% phosphate-buffered formalin solution at room temperature for 2 days. They were then decalcified in 10% ethylenediaminetetraacetic acid solution (EDTA solution, pH 7.0, Fujifilm, Tokyo, Japan) at room temperature for 4 weeks. When the samples were trimmed, the three axes and regions of interest (ROIs) were designated (Fig. 1) [22]. The reference axes of the specimens were designated as follows. The *X*-axis was defined as the extension of the line joining the lowest point of the thickened area of the anterior mandible (a) and the lowest point of the thickened area of the posterior mandible (p), and the *Z*-axis as the axis perpendicular to the *X*-axis on the plane passing through points a, a’, p, and p’ (the XZ plane). The *Y*-axis was defined as the axis orthogonal to these two axes. The decalcified specimens were embedded in paraffin by the conventional method, and 7-µm-thick, consecutive thin slices passing through the center of the mesial root apex (Section A), the center of the buccolingual root apex (Section B), and the center of the distal root apex (Section C) of M1 in planes parallel to the YZ plane were prepared. These sections were stained with hematoxylin–eosin (H–E) for morphological observation, after which immunohistochemical staining was conducted with anti-S100 antibodies for observation of myelin sheath in myelinated nerves and with anti-neurofilament light polypeptide (NF-L) antibodies for observation of axons. Tissue section observations were made with a universal optical microscope (Axiophot 2; Carl Zeiss, Oberkochen, Germany).

### Immunohistochemical staining

Immunohistochemical analysis was performed on 7 μm-paraffin sections of the rat mandibles on 1, 3, 5 and 7 day post-extraction. Paraffin-embedded sections were deparaffinized and treated with 0.3% H2O2-containing solution at room temperature for 30 min to inactivate the endogenous peroxidase, blocked with 10% bovine serum albumin (A9647; Sigma-ALDRICH Inc, St. Louis, Missouri, USA) at room temperature for 60 min, and then reacted with a primary antibody. A rabbit polyclonal anti-S100 protein (S100) antibody (ab34686; Abcam, Cambridge, UK) (1:1000 dilution) was used as the primary antibody for myelin sheath. A rabbit polyclonal anti-neurofilament light polypeptide (NF-L) antibody (ab238420; Abcam, Cambridge, UK) (1:1000 dilution) was used as the primary antibody for axon. The sections were reacted with the primary antibodies at 4℃ for overnight. As a secondary antibody, Avidin–Biotinylated Enzyme Complex (VECTASTAIN Elite ABC Kit: VECTOR LABORATORIES, Newark, California, US) was used for the subsequent procedures. Both sections, after three rinsing steps with PBS, the sections were incubated with biotin-labelled anti rabbit goat IgG, following after three rinsing steps with PBS, after which they were incubated with peroxidase-labelled streptavidin. Next, after three rinsing steps with PBS, the sections were treated with color development was activated using DAB (ImmPACT® DAB; VECTOR LABORATORIES, Newark, California, US). Finally, counterstaining was performed using hematoxylin. Tissue section observations were made with a universal optical microscope (Axiophot 2; Carl Zeiss, Oberkochen, Germany).

### Evaluation of the nerve regeneration

To quantitatively evaluate the nerve regeneration process after tooth extraction, the cross-sectional area of the myelinated nerve at each stage (non-extraction ~ 7 day post-extraction) was compared. Using photographs taken under a × 40 objective lens, the cross-sectional area of the target myelinated nerve, the area positive for anti-NF-L and S-100 antibodies were traced and scanned using Adobe Illustrator CC. To calculate These areas of the traced in the visual field, the scanned images were processed using IMAGE-PRO®PLUS (Media Cybernetics, Inc., Rockville, Maryland, USA).

### Statistical analysis

Tukey’s multiple comparison test was performed after the percentage of positive area for anti-NF-L and S-100 antibodies to the total cross-sectional area of the myelinated nerve were calculated. *P* values of < 0.05 were considered to indicate high statistical significance. Data were analyzed with the SPSS 18.0 statistical software package (SPSS, Chicago, Illinois, USA).

## Results

### Histological and immunohistochemical observations

**Fig. 2:**
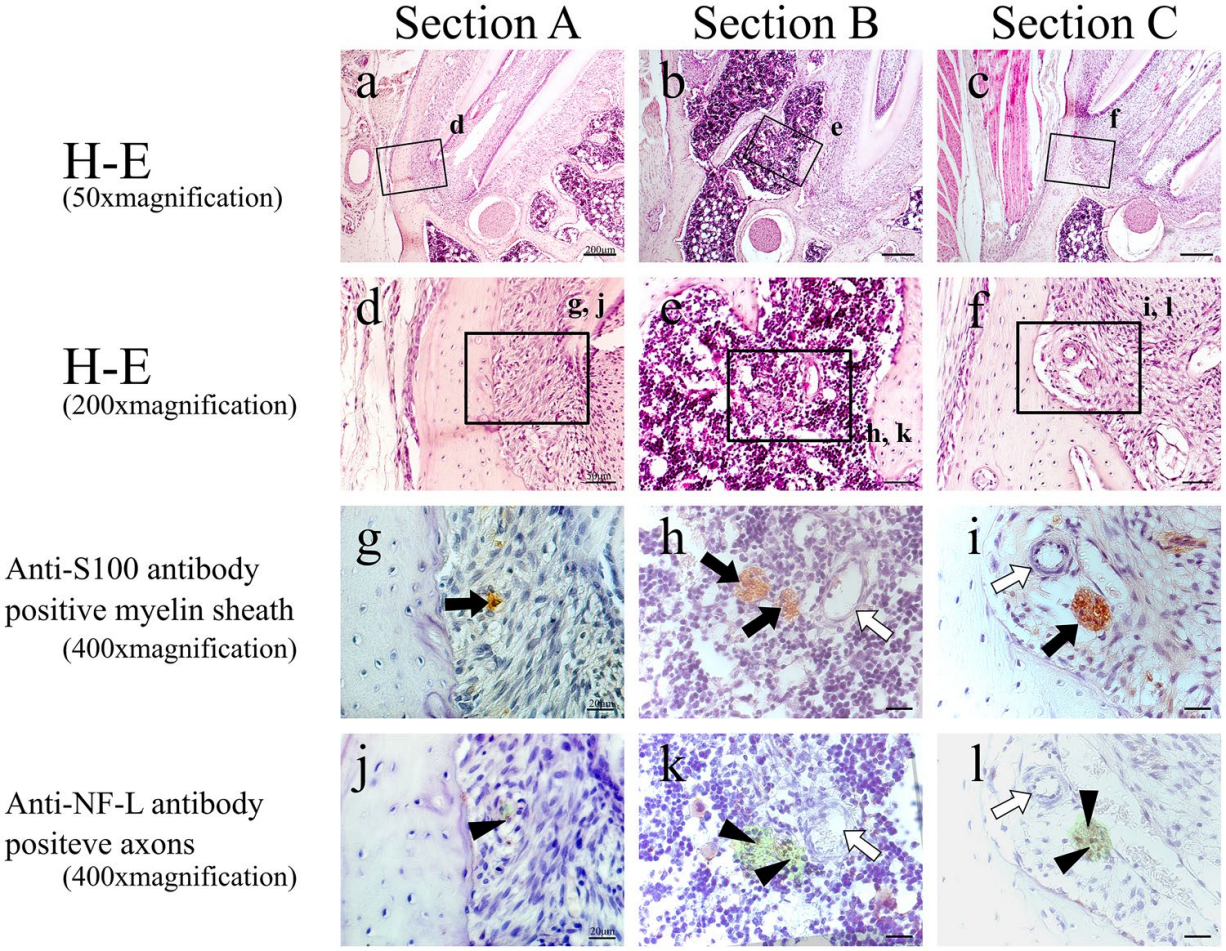
H–E-stained specimens (**a**–**c** 50 × magnification; **d–f** 200 × magnification) and anti-S100 antibody (**g**–**i** 400 × magnification) and anti-NF-L antibody (**j**–**l** 400 × magnification)-immunostained specimens of each cross section in the control group. Green region means serial-sectional S100-positive location (**j**–**l**). Black arrow: myelin sheath (S100 protein marker positive); white arrow: blood vessel; black arrow head: axons (NF-L marker positive)

H–E-stained specimens and anti-S100 antibody and anti-NF-L antibody–immunostained specimens of each cross section in the control group are shown in Fig. 2. In Section A, the alveolar branch was present in the apical periodontal tissue at the mesial root (Fig. 2a, d), in Section B, it was present in the bone marrow between the buccal and lingual roots (Fig. 2b, e), and in Section C, it was present in the apical periodontal tissue at the distal root (Fig. 2c, f). The alveolar branch observed in all these sections expressed the S100 protein, a marker for myelin sheath (Fig. 2g–i), and NF-L, a marker for axons, was also evident within it (Fig. 2j–l), demonstrating that it consisted of a bundle of myelinated nerves.

**Fig. 3:**
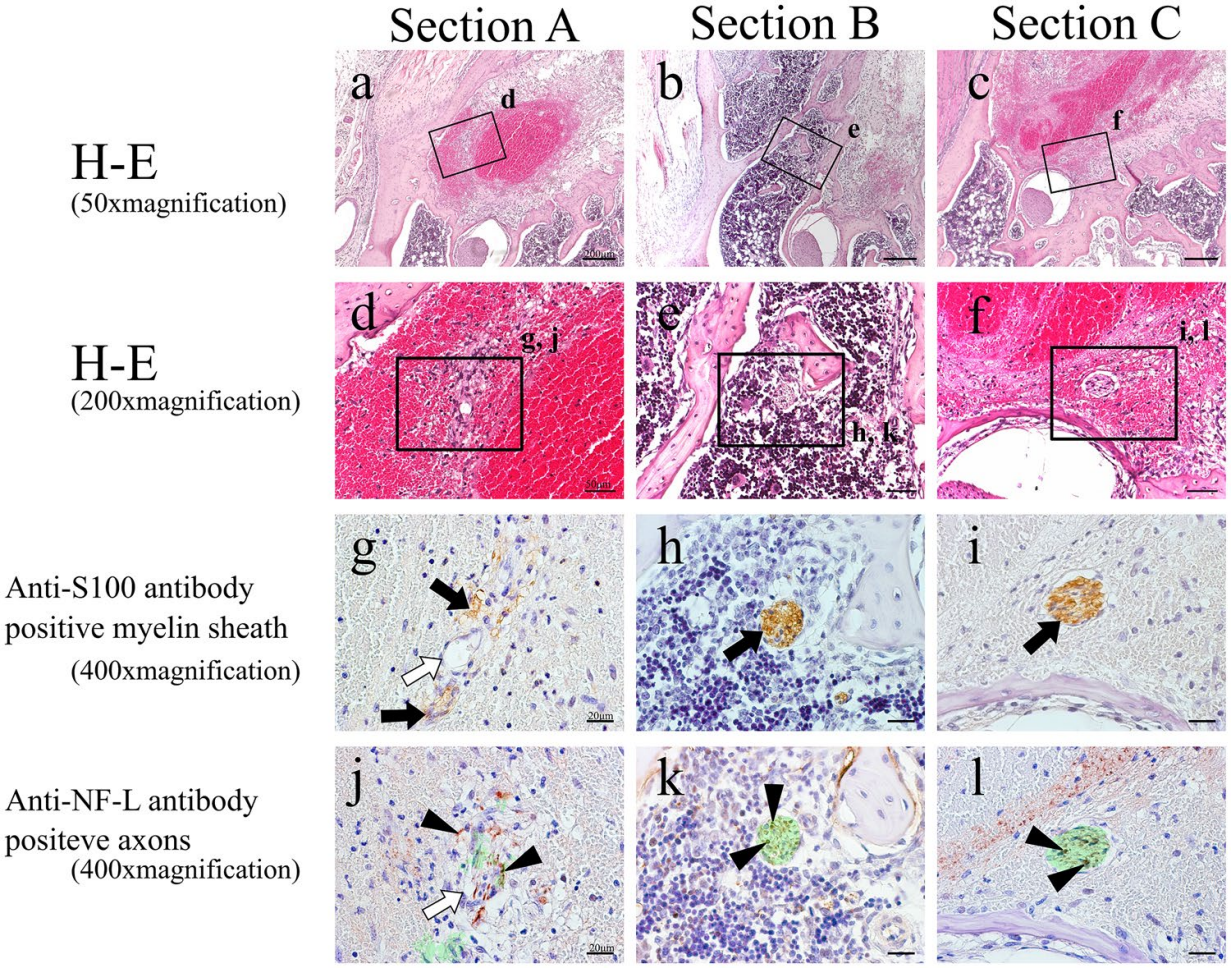
H–E-stained specimens (**a**–**c** 50 × ; **d**–**f** 200 × magnification) and anti-S100 antibody (**g**–**i** 400 × magnification) and anti-NF-L antibody (**j**–**l** 400 × magnification)-immunostained specimens of each cross section from day 1. In Section A, the myelin sheaths and axons have melted away, but this is not evident in Sections B and C. Green region means serial-sectional s100-positive location (**j**–**l**). Black arrow: myelin sheath; white arrow: blood vessel; black arrow head: axons

**Fig. 4:**
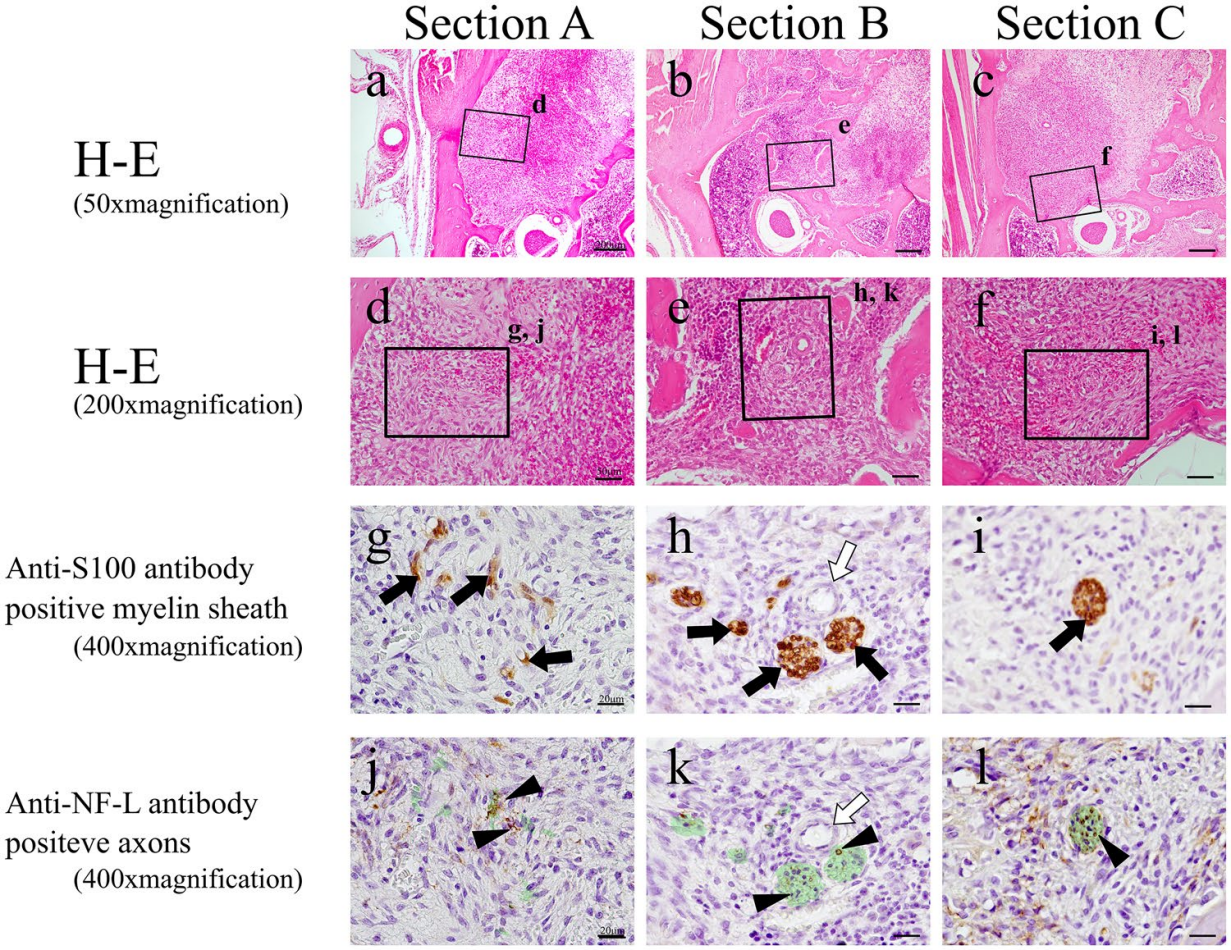
H–E-stained specimens (**a**–**c** 50 × ; **d**–**f** 200 × magnification) and anti-S100 antibody (**g**–**i** 400 × magnification) and anti-NF-L antibody (**j**–**l** 400 × magnification)-immunostained specimens of each cross section from day 3. In Section A, scattered myelin sheaths themselves are present, and axons are evident across a wider extent of the extraction socket than the myelin sheaths. In Sections B and C, the distributions of the axons are very similar to those of the myelin sheaths. Green region means serial-sectional S100-positive location (**j**–**l**). Black arrow: myelin sheath; white arrow: blood vessel; black arrow head: axons

**Fig. 5:**
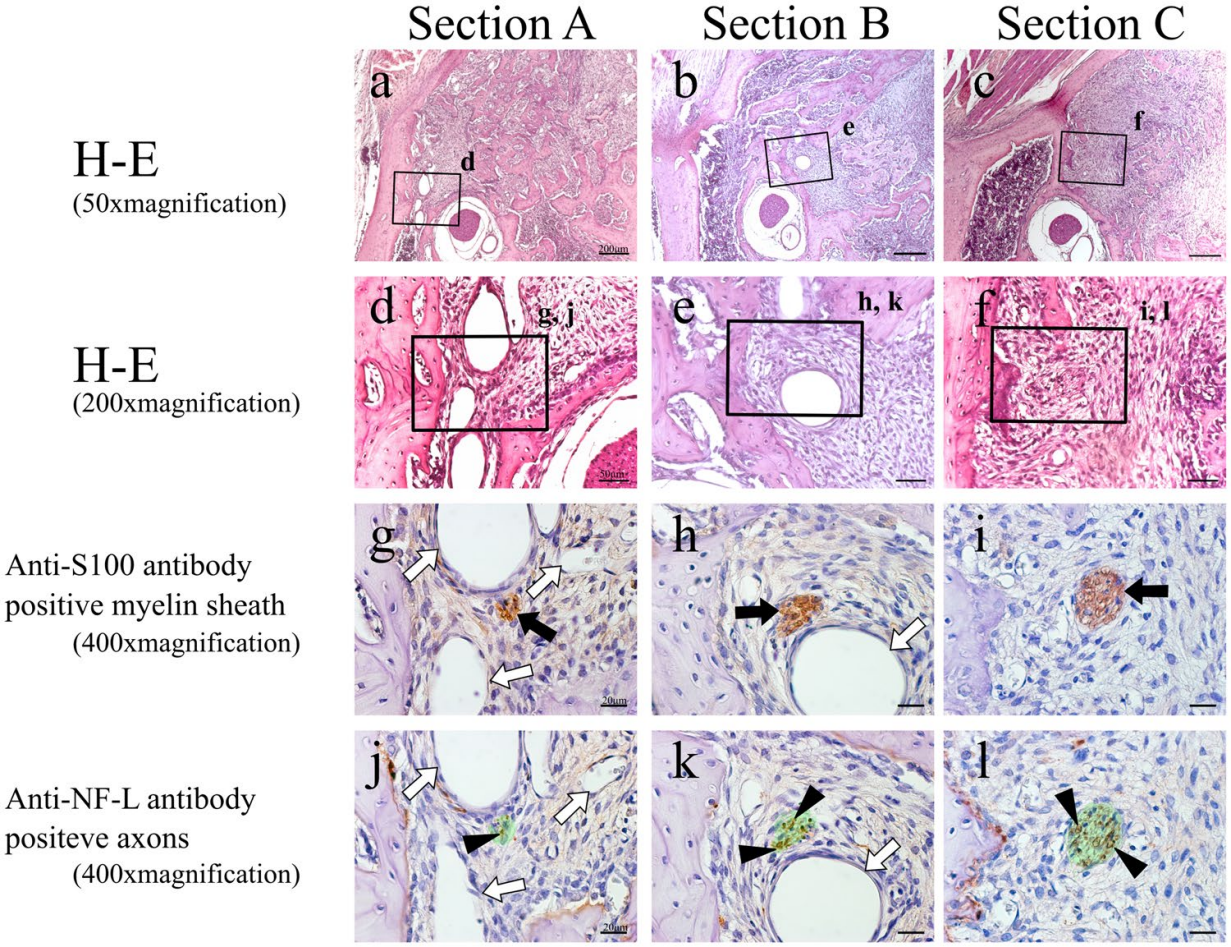
H–E-stained specimens (**a**–**c** 50 × ; **d**–**f** 200 × magnification) and anti-S100 antibody (**g**–**i** 400 × magnification) and anti-NF-L antibody (**j**–**l** 400 × magnification)-immunostained specimens of each cross section from day 5. In Sections A and B, marked dilation of the blood vessels is evident and nerves are also present in the vicinity in association with the vessels, but this is not evident in Section C. Green region means serial-sectional S100-positive location (**j**–**l**). Black arrow: myelin sheath; white arrow: blood vessel; black arrow head: axons

**Fig. 6:**
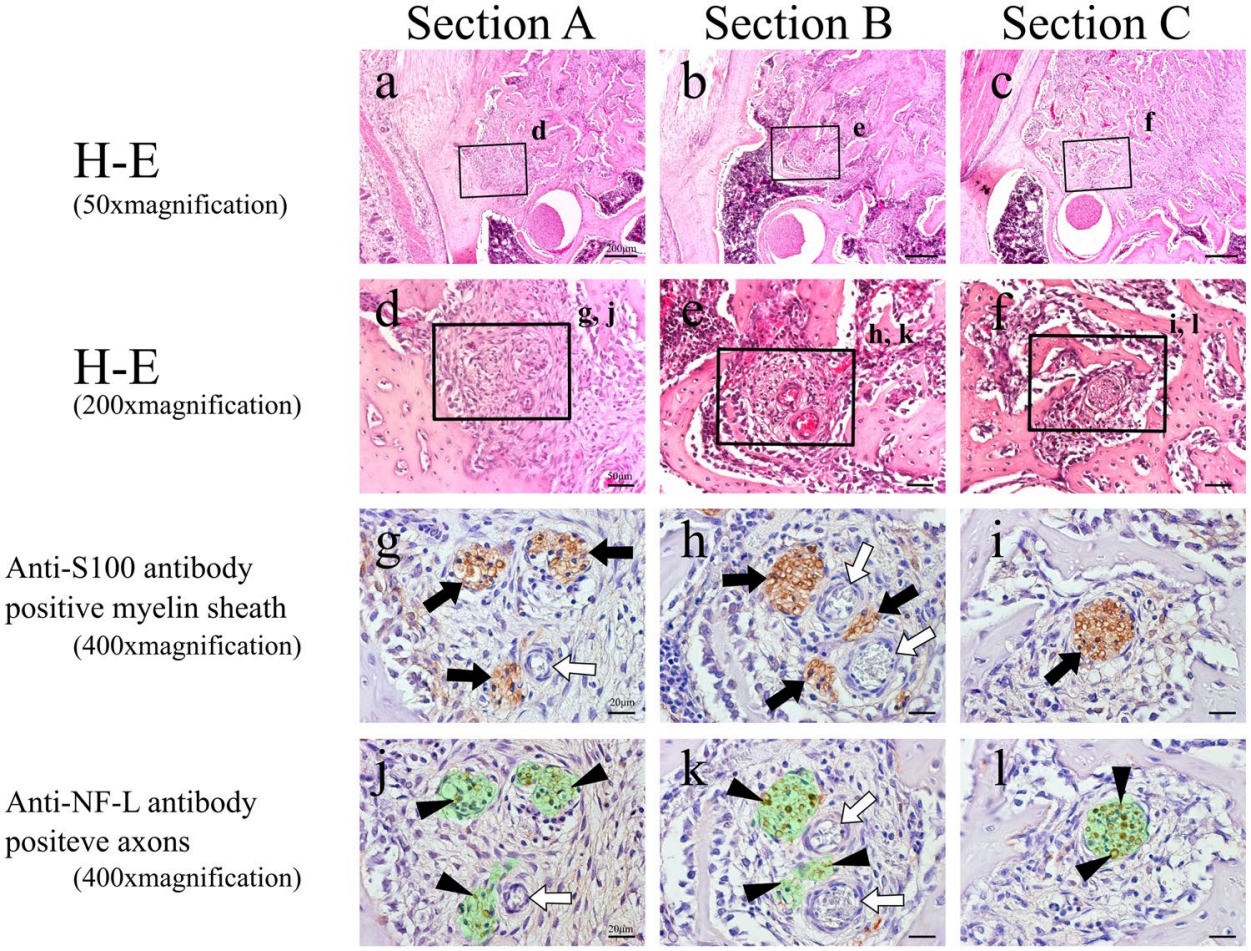
H–E-stained specimens (**a**–**c** 50 × ; **d**–**f** 200 × magnification) and anti-S100 antibody (**g**–**i** 400 × magnification) and anti-NF-L antibody (**j**–**l** 400 × magnification)-immunostained specimens of each cross section from day 7. Recovery from the extraction is very apparent, and in Sections B and C, bone remodeling is evident in the vicinity of the nerves, while in Section A, the distribution of the myelin sheaths and axons displays a similar histology compared with those on days 3 and 5. Green region means serial-sectional S100-positive location (**j**–**l**). Black arrow: myelin sheath; white arrow: blood vessel; black arrow head: axons

On post-extraction day 1, no alveolar branch was evident within the blood clot, and the nerve in the apical region had been severed by the extraction (Fig. 3a, d). The areas where S100 protein and NF-L were expressed were botted and not completely consistent with each other. In Sections B and C, located distally to Section A, an alveolar branch was visible within the bone marrow (Fig. 3b, c, e, f). This alveolar branch expressed both S100 protein (Fig. 3h, i) and NF-L (Fig. 3k, l), showing that it was a similar bundle of myelinated nerves to that seen in the control group. On post-extraction day 3, enlarged blood vessels was evident with myelinated nerve in granulation tissue mixed with fibroblasts and inflammatory cell (Fig. 4a-–f). In Section A, no nerve bundle forming an alveolar branch was observed in the granulation tissue in the apical region (Fig. 4a, d). The expressions of S100 protein and NF-L in this section were even less coherent than on post-extraction day 1 (Fig. 4g, j). In Sections B and C, an alveolar branch was evident in the bone marrow, which also contained granulation tissue. Microvessels were also observed in the vicinity of the alveolar branch (Fig. 4e, f). This alveolar branch expressions of S100 protein by this alveolar branch were similar to those seen in the control group (Fig. 4h, i), but expression of NF-L was greatly diminished compared with that on post-extraction day 1 (Fig. 4k, l). On post-extraction day 5, a small nerve bundle attached to newly generated blood vessels between the fibrous bone was evident in Section A. In this nerve bundle, part of an alveolar branch followed the course of a vessel to enter the mesial root extraction socket. This nerve bundle expressed S100 protein, and a small amount of NF-L expression was evident in its interior (Fig. 5g, j). In Section B, an alveolar branch was evident between the formed fibrous bone, with blood vessels running in its vicinity (Fig. 5b, e). This alveolar branch expressed NF-L in the interior of S100 protein, but compared with the control group, there were fewer NF-L-positive axons (Fig. 5h, k). In Section C, an alveolar branch was present in the connective tissue (fibrous layer) between the fibrous bone (Fig. 5c, f). This alveolar branch expressions of S100 protein by this alveolar branch were similar to those seen in the control group, but expression of NF-L was increased on day 3, but less than in the control group (Fig. 5i, l). On post-extraction day 7, bone formation had reached the alveolar crest (Fig. 6). Multiple branched nerve bundles were evident at the bottom of the extraction socket in Section A (Fig. 6a, d). In Sections B and C, an enlarged alveolar branch was observed and the vessels in its vicinity had contracted (Fig. 6e, f) and these nerve bundles expressed s100 protein (Fig. 6h, i). However, NF-L expression in this enlarged alveolar branch was weaker than control group (Fig. 6k, l).

In summary, the following were the three main findings of this experiment. (1) The only obvious nerve damage caused by extraction was to the alveolar branch close to the mesial root (Section A), and axons deviating from the myelin sheath were observed. (2) On the central side of the mesial root (Sections B and C), although there was no destruction of the myelin sheath, the axons inside it tended to be fewer in number. (3) Finally, myelinated nerve regeneration took place in the vicinity of dilated new blood vessels, and it was particularly pronounced in Section A on day 5 post-extraction. These findings suggested that damage to the myelinated nerve caused the degeneration of both the axons and the myelin sheath at the periphery (Section A), but that myelin sheath degeneration did not occur more centrally to this (Sections B and C). This is why the rearrangement of axons occurred on the foundation of the residual myelin sheaths on the central side, and peripheral nerves were regenerated in parallel to the blood vessels newly generated in the process of tissue restoration.

### Evaluation of the nerve regeneration

**Fig. 7:**
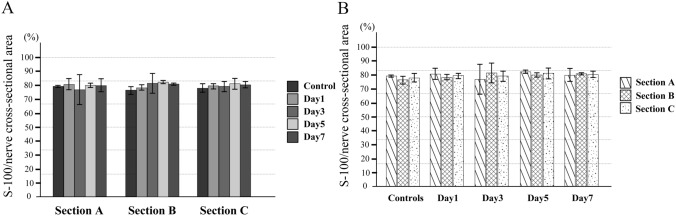
Proportions of myelin sheaths in nerve fiber cross section. There is no significant difference in the proportion of myelin sheaths between the control and experimental groups. **A** Comparison for each phase; **B** comparison for each area

**Fig. 8:**
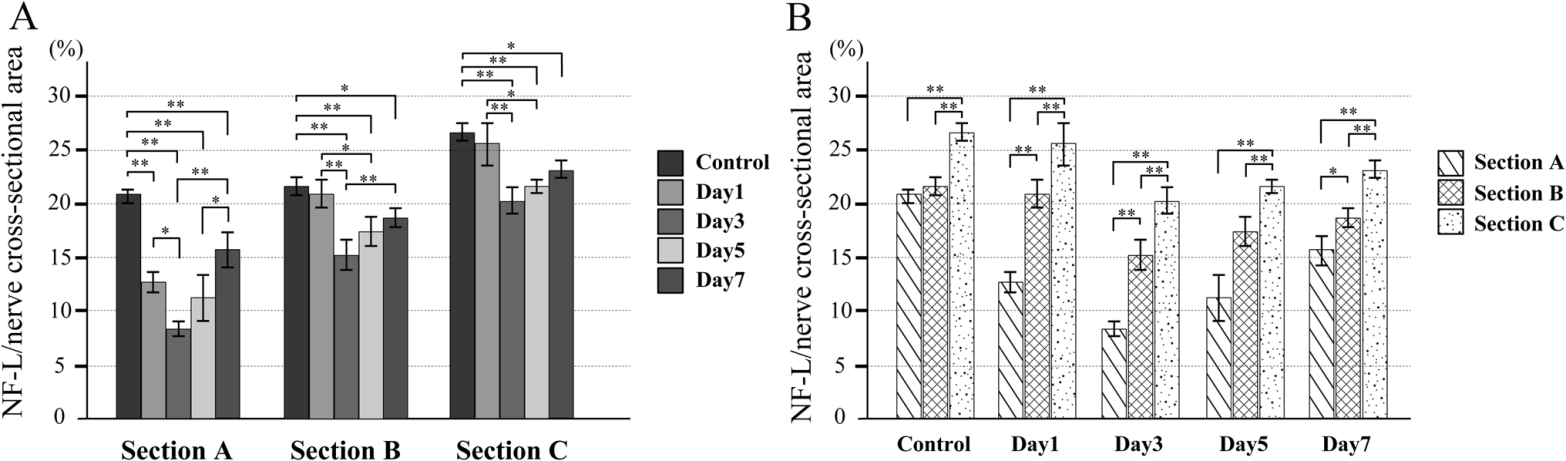
Proportions of axons in nerve fiber cross section. On day 1, the proportion of axons in Section A is significantly lower in the experimental group, although there is no significant difference with the control group in Sections B and C. On day 3, the axons have also disappeared from Sections B and C, and their proportion is significantly lower than that of the control group in all three sections. Then, on day 5, the proportion of axons increases in Sections A, B, and C. In all three sections, however, the proportions are significantly lower than in the control group on the same day. The results for day 7 are similar to those for day 5. **A** Comparison for each phase; **B** comparison for each area

There is no significant difference in the proportion of myelin sheaths between the control and experimental groups at every phase and in all cross sections (Fig. 7). Figure 8 shows the proportions of axons in the nerve fiber cross sections at each ROI. On day 1, there was also no significant difference between the proportions of axons in all nerve fiber section of the control group. However, on day 1, the proportion of axons in Section A was significantly lower in the experimental group, although there was no significant difference with the control group in Sections B and C. On day 3, the axons had disappeared from Sections B and C as well, and their proportion was significantly lower than of the control group in all three sections. Then, on day 5, the proportion of axons increased in Sections A, B, and C, with the highest level seen in Section C, and Sections B and A having significantly lower values, in that order. In all three sections, however, the proportions were significantly lower than in the control group on the same day. The results for day 7 were similar to those for day 5.

**Fig. 9:**
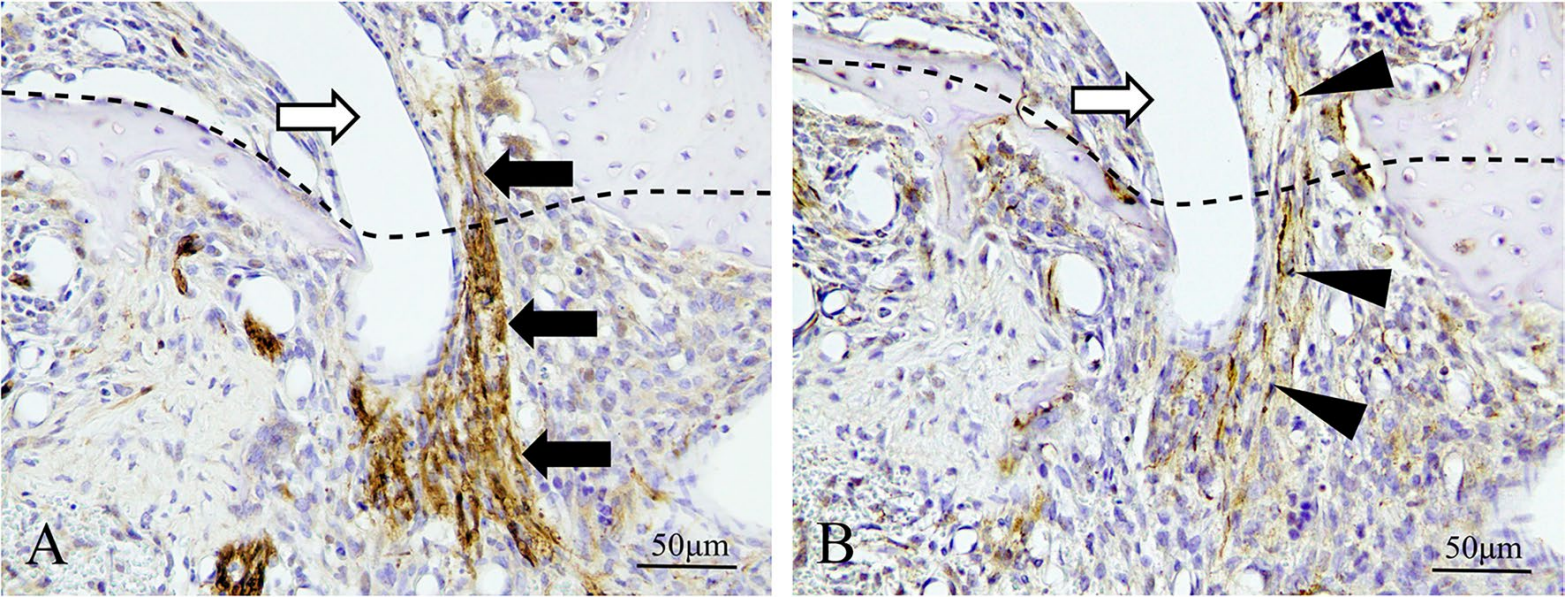
Horizontal sectional images of boundary between extraction socket and alveolar bone on day 3 (400 × magnification). **A** Anti-S100 antibody immunostained image; **B** anti-NF-L antibody immunostained image. There is entry by myelinated nerves into the extraction socket. Above the dotted line is bone marrow area, below the line is extraction socket area. Black arrow: myelin sheath; white arrow: blood vessel; black arrow head: axons

**Fig. 10:**
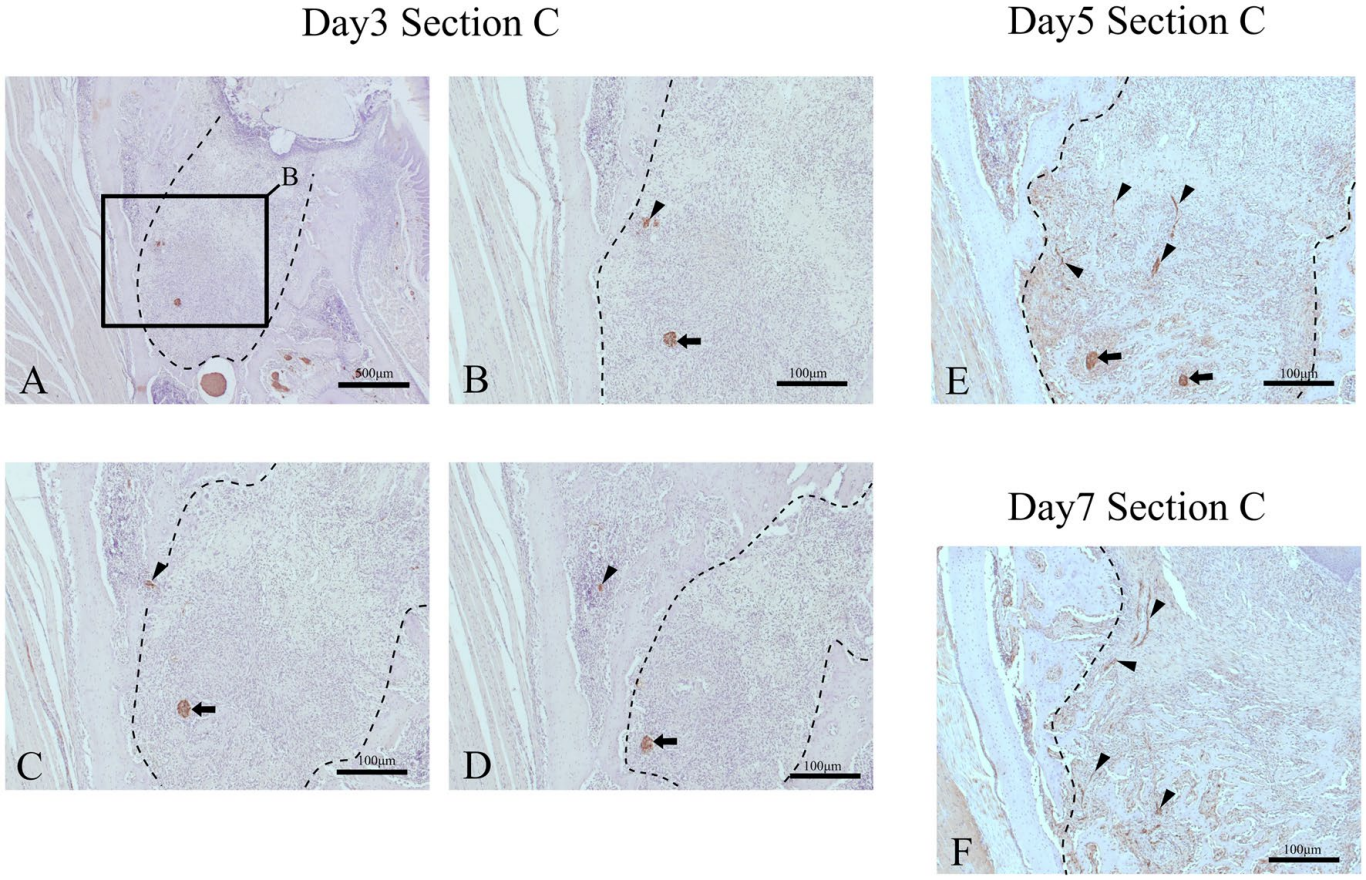
Cross-sectional images of myelinated nerve-stained anti-S100 antibody entering the extraction socket. On day 3, there is slight entry by myelinated nerves into the extraction socket. On day 5, there are signs of the entry of myelinated nerves into the extraction socket in Section C. On day 7, myelinated nerves entering the extraction socket from the lateral surface are evident in Section C. **A**–**D** Serial sections of extraction socket and surrounding alveolar bone at Section C on day 3. **E** A section of extraction socket at Section C on day 5. **F** A section of extraction socket at Section C on day 7. Black arrow: alveolar branch (S100 protein marker positive); black arrow head: invading myelinated nerve in extraction socket (S100 protein marker positive); dotted line: extraction socket

Figure 9 shows horizontal sectional images of myelinated nerve entering the extraction socket. There is entry by myelinated nerves into the extraction socket on day 3. A continuous section showed that the myelinated nerve was located near the alveolar bone on the buccal side as it migrated to the central side (Fig. 10A–D). On post-extraction day 5, many fine nerves were found in the extraction socket, extending from the lateral surface of bone marrow (Fig. 10E). On post-extraction day 7, the number of nerves in the extraction socket increased (Fig. 10F).

## Discussion

Hansen conducted a study of nerve injury due to extraction and its healing by investigating the post-extraction retrograde response of the trigeminal ganglion and axonal degeneration in the extraction socket over time [23]. According to that study, the first retrograde response was evident in the cell bodies of the trigeminal ganglion 12 h after extraction, and this response peaked at 1 week post-extraction, before returning to its normal state after 3 weeks. Hansen also carried out a study of nerve tissue regeneration in the extraction socket, finding that, on post-extraction day 4, new axon regeneration was evident within the blood clot, and that, on post-extraction day 6, long, thin axons were extending within the blood clot. The response in the trigeminal ganglion is consistent with all the subsequently published papers that have described the effect of extraction on the cell bodies of the trigeminal ganglion [13, 23]. They reported that axons are evident in the extraction socket after a few days, and suggested that although the myelinated nerve fibers that innervate the tooth pulp and periodontal ligament undergo retrograde degeneration as a result of tooth extraction, after some days, they are regenerating and extending at the alveolar branch level. In the present study, the presence of nerve fiber bundles in the blood clot was already evident on post-extraction day 3, and on post-extraction day 7, similar results were obtained. Detailed observations of the alveolar branches in the area corresponding to the apex further showed that, on day 3, the number of axons in Sections B and C had greatly decreased, indicating that, after extraction, the connection between peripheral nerve tissue and the trigeminal ganglion was temporarily markedly reduced in the region of the alveolar branch. Subsequently, although the myelin sheaths were regenerating on day 5, the majority of the axons of the alveolar branches extending from the inferior alveolar nerve were seen to be extremely thin and scattered, despite their further regeneration. This was considered appropriate from the viewpoint of the previously reported speed of axon regeneration [24]. Furthermore, because the organs (the tooth and periodontal tissue) to which the axons that had innervated the tooth and its surrounding periodontal tissue should have been connected had been lost as a result of extraction, nerve regeneration did not actively take place in the direction of the extraction socket even if myelin sheaths were still present [25]. That is, the nerve fiber bundles that Hansen observed appearing in the extractions socket at an early stage were probably not axons extending from the alveolar branch in the apical region, but were rather derived from myelinated nerves that had been directly innervating the bone marrow, a finding that is extremely interesting. The various transmitter substances associated with nerve regeneration are known to have a major effect on extraction socket healing, but if the axons that are thought to be directly associated with extraction socket healing are actually derived from the bone marrow of surrounding alveolar bone, this discovery would have a major effect on the treatment strategy for promoting extraction socket healing.

## Conclusions

The present results suggest that the axon regeneration previously reported to take place at an early stage in the extraction socket probably consisted of extensions from the surrounding bone marrow, rather than being derived from the alveolar branches that had innervated the extracted tooth. It was also shown that the axonal extension from the bone marrow does not reach through the entire extraction socket, which becomes alveolar bone similar to normal cancellous bone with a few sensory nerves.

## Data Availability

The datasets generated and/or analyzed during the current study are available from the corresponding author on reasonable request.
